# An observational naturalistic study on non-suicidal self-harm behaviours in a cohort of adolescents and young inpatients during COVID-19 outbreak

**DOI:** 10.1192/j.eurpsy.2022.656

**Published:** 2022-09-01

**Authors:** L. Orsolini, E. Ribuoli, E. Fiordelmondo, C. Appignanesi, L. Martino, V. Salvi, U. Volpe

**Affiliations:** Polytechnic University of Marche, Department Of Clinical Neurosciences/dimsc, School Of Medicine, Unit Of Psychiatry, Ancona, Italy

**Keywords:** Self Harm, NSSI, Covid-19, adolescent

## Abstract

**Introduction:**

Non-suicidal self-harm (NSSH) include deliberate behaviours with the intent to self-injure. NSSH prevalence ranges 15.5%-31.3% in adolescents and young adults<25 years-old.

**Objectives:**

Our aim is characterizing the psychopathological domains occurring in adolescent and young adults with NSSH during the second COVID-19-related wave (October 2020-August 2021).

**Methods:**

A cross-sectional study recruited inpatients aged 15-24 consecutively afferent to psychiatric ward due to NSSH, by investigating anger rumination(ARS), emotional regulation (DERS), dissociation (DES-II), metacognitive capabilities(MCQ-30), perceived stress (PSS), self-criticism (LOSCS), emotional intelligence (Reading the Mind in the Eyes Test-RMET), aggressiveness (AQ), impulsiveness (BIS-11), hopelessness(BHS), alexithymia (TAS-20). NSSH were characterized by using suicide score scale(SSS) and deliberate self-harm interview (DSHI).

**Results:**

A 7-fold increase in young inpatient access was observed from 2019 to 2021. DSHI median was 2 (95%CI=1,17-2,73), SSS-12months median was 5 (95%CI=4.2-6.7), SSS-lifetime median was 5 (95%CI=3.4-5.3) and MINI median was 5 (95%CI=3.4-4.7). Linear regression analysis and Pearson’s correlations revealed strong correlations between DSHI and BHS (r=0.550), TAS-20 (r=0.495), AQ-hostility(r=0.529),AQ-total (r=0.446), PSS(r=0.454), DERS-total (r=0.621), DERS-lack_of_control (r=0.658),MCQ-total(r=0.534),MCQ-perception_danger_not_ control (r=0.583); between SSS-12months and AQ-total (r=0.456), AQ-Anger (r=0.443), BIS-total(r=0.457),BIS-Attentional-Impulsiveness (r=0.511),BIS-Complex-Motor-Impulsiveness (r=0.507), PSS (r=0.617), DERS(r=0.571), DES(r=0.559).

**Conclusions:**

COVID-19-related increased perceived stress and depressive symptomatology may have facilitated the onset of severe NSSH in adolescents and young people with trait impulsiveness, hostility and affective dysregulation.

**Disclosure:**

No significant relationships.

